# Editorial: Innate lymphoid cells in cancer: volume II

**DOI:** 10.3389/fimmu.2026.1891880

**Published:** 2026-06-15

**Authors:** Nicolas Jacquelot, Emilie Narni-Mancinelli, Alexander David Barrow

**Affiliations:** 1 Department of Biochemistry and Molecular Biology, Cumming School of Medicine, University of Calgary, Calgary, AB, Canada; 2 Department of Microbiology, Immunology and Infectious Diseases, Cumming School of Medicine, University of Calgary, Calgary, AB, Canada; 3 Riddell Centre for Cancer Immunotherapy, Arnie Charbonneau Cancer Institute, Arthur J.E. Child Comprehensive Cancer Centre, University of Calgary, Calgary, AB, Canada; 4 Alberta Children’s Hospital Research Institute, University of Calgary, Calgary, AB, Canada; 5 Calvin, Phoebe, and Joan Snyder Institute for Chronic Diseases, University of Calgary, Calgary, AB, Canada; 6 Aix Marseille Universite, INSERM, CNRS, Centre d’Immunologie de Marseille-Luminy, Marseille, France; 7 Department of Microbiology and Immunology, The University of Melbourne and The Peter Doherty Institute for Infection and Immunity, Melbourne, VIC, Australia

**Keywords:** cancer, immunotherapy, innate immunity, innate lymphoid cells, natural killer cells, γδ T cells

The second volume of the Community Series on Innate Lymphoid Cells (ILCs) in Cancer arrives at a pivotal moment in immunological research, as the boundaries between innate and adaptive immunity continue to blur, and the tumor microenvironment (TME) is increasingly recognized as a dynamic ecosystem of cellular interactions. Collectively, the articles published in this volume highlight the expanding role of ILCs, not just as passive tissue responders, but as active modulators of cancer progression and therapeutic outcomes.

ILCs comprise five distinct subsets―NK cells, ILC1, ILC2, ILC3, and Lymphoid Tissue inducer (LTi) cells―that mirror the functional diversity of adaptive T cells while operating independently of somatically rearranged antigen-specific receptors. Their rapid responsiveness and potent cytokine-producing capacity position them as key regulators of tissue homeostasis and inflammation (1). In cancer, this duality becomes a double-edged sword. While NK cells are predominantly anti-tumorigenic, the roles of so-called helper ILC subsets are more nuanced and context-dependent.

This volume brings together cutting-edge research exploring how ILCs interact with the TME, influence immunosurveillance, and respond to immunotherapeutic interventions. The contributions underscore the remarkable plasticity of ILCs, their susceptibility to tumor-derived signals, and their capacity to either restrain or promote tumor growth depending on tumor type and stage. Such findings challenge the traditional paradigms of immune cell behavior and open potential new avenues for ILC-based therapeutic strategies.

The series emphasizes the need for a deeper understanding of ILC phenotype, function and regulatory mechanisms, including their expression of immune checkpoints and cytokines. Seo et al. contributed an important technical advance to the field by developing a 25-parameter spectral flow cytometry panel optimized for the high-resolution discrimination of ILC subsets within mammary tumors. Their approach combines detailed lineage exclusion strategies with transcription factor-based profiling and is supported by an accompanying computational pipeline enabling unbiased clustering and phenotypic analysis. Importantly, the workflow performs reproducibly across two distinct spectral cytometers, underscoring its reliability and broad applicability. This approach provides a valuable resource for routine and high-fidelity characterization of rare ILC populations in solid tumors. Sakata et al. demonstrated that immunoglobulin-like transcript 2 (ILT2, also known as LILRB1) is upregulated on CD56^dim^ NK cells in hepatocellular carcinoma (HCC), where it is associated with impaired cytotoxicity. This identifies ILT2 as a key innate immune checkpoint shaped by aging and the TME. These insights are crucial for designing strategies that can rewire ILC functions toward anti-tumor immunity without exacerbating inflammation or autoimmunity. Maddineni et al. identified a population of IL-13-producing intraepithelial ILC1-like CD56^dim^ NK cells in Head and Neck Squamous Cell Carcinoma (HNSCC) tumors, revealing a cytokine-driven phenotype that may contribute to tumor progression. The review by Zhu et al. provides a comprehensive overview of γδ T cell subsets, describing their phenotypes and function in cancer. They emphasize on the therapeutic potential of these innate-like T cell subsets and their capacity to be co-opted for cancer treatment. Specifically, this article underscores the potential synergy between γδ T cells and NK cells, particularly in enhancing anti-tumor responses through immune checkpoint modulation and CAR-based therapies. Finally, the study by Khan et al. shows that CD56^bright^ NK cells may be the dominant NK subset infiltrating bladder tumors, and their transcriptional signature is associated with favorable patient prognosis. The CD56^bright^ NK cells transcriptional signature is associated with activating receptors and tissue-residency markers and correlated with a dendritic cell and CD8^+^ effector memory T cell signatures, suggesting potential crosstalk between CD56^bright^ NK cells, dendritic cells and CD8^+^ T cells driving anti-tumor immunity.

As the field evolves, the integration of ILC biology into cancer immunotherapy holds considerable promise for the development of more precise and effective therapeutic approaches. This volume not only reflects the current state of knowledge but also sets the stage for future exploration into the therapeutic manipulation of ILCs.
